# Prenatal detection of Peters plus-like syndrome

**DOI:** 10.4274/tjod.45649

**Published:** 2019-01-09

**Authors:** Mehmet Tunç Canda, Latife Doğanay Çağlayan, Ayşe Banu Demir, Namık Demir

**Affiliations:** 1 Kent Hospital, Clinic of Obstetrics and Gynecology, İzmir, Turkey; 2 Kent Hospital, Laboratory of Clinic Pathology, İzmir, Turkey; 3 İzmir Economics University Faculty of Medicine, Department of Medical Biology, İzmir, Turkey

**Keywords:** Peters anomaly, Peters plus syndrome, prenatal diagnosis, congenital cataract, B3GALTL gene

## Abstract

Peters plus syndrome is a rare congenital disorder that includes ocular anterior segment defects of the classic Peter’s anomaly, and is mostly associated with craniofacial and skeletal defects. A 21-week fetus was referred for further evaluation due to a suspicion of fetal hydrocephalus. An ultrasound examination revealed hyperechogenic lenses, microphthalmia, hypotelorism, retrognathia, mild ventriculomegaly, absence of the cavum septum pellucidum, and short stature. Amniocentesis and further microarray analysis revealed normal chromosomal copy numbers including the gene *B3GALTL*. In utero mort fetalis occurred at the 23^rd^ gestational week. Ultrasound and fetal autopsy findings were suggestive of Peters plus syndrome, but the absence of the *B3GALTL* gene mutation made the diagnosis Peters plus-like syndrome. Obstetricians should consider Peters plus-like syndrome with prenatal detection of ocular anomalies along with craniofacial and skeletal anomalies with the absence of *B3GALTL* gene mutation.

## Introduction

Peters’ anomaly is a rare congenital ocular anomaly caused by defective dysgenesis and cleavage of the anterior chamber of the eye causing central corneal opacity (leukoma), absence of the posterior corneal stroma and Descemet membrane, and a variable degree of iris and lenticular attachments to the central aspect of the posterior cornea^(1)^. Peters plus syndrome, previously known as Krause-Kivlin syndrome or Peters’ anomaly with short-limb dwarfism (OMIM ≠ 261540), is an autosomal-recessive inherited congenital disorder caused by a mutation in the *B3GALTL* gene on chromosome 13q12.3. Peters plus syndrome is a rare anomaly with unknown incidence, with equal sex ratio, and a high incidence of consanguinity. Just over 70 cases have been reported in the postnatal period and 8 cases in the prenatal period^(2,3,4,5,6)^. The classic triad of Peters plus syndrome includes anterior segment defects (100%), short stature (100%), and brachydactyly (95%)^(7)^. There are also Peters plus-like syndromes, which did not carry a mutation in the *B3GALTL* gene, but have similar anomalies to the classic Peters-plus syndrome^(8)^.

Herein, we report the prenatal diagnosis of Peters plus-like syndrome in a Turkish family without the *B3GALTL* gene mutation.

## Case Report

**Figure 1:**
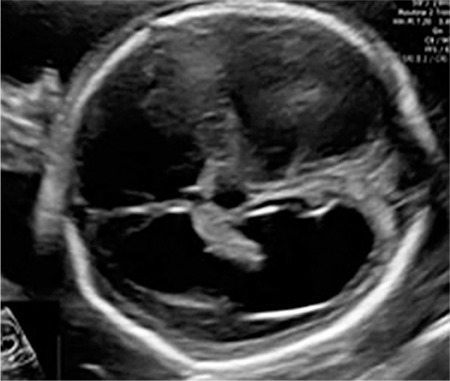
Hyperechogenic patterns of the lens and anterior chamber

**Figure 2:**
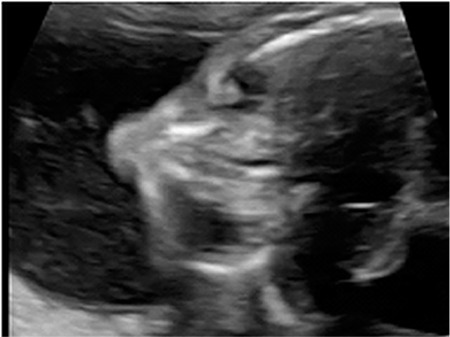
Binocular distance was 29 mm (5^th^ percentile), inter-ocular distance was 13.5 mm (50^th^ percentile) and ocular diameter 7.6 mm (<5^th^ percentile) (microphthalmia) at 21 weeks

A 23-year-old woman, gravida1 partus 0, was referred to our clinic at the 21^st^ week of her pregnancy due to the suspicion of fetal hydrocephalus. The fetal biometry scan showed appropriate biometric measurements, but long bone measurements, including femur, fibula, radius, and ulna were one week shorter than the expected gestational age. The detailed scan showed a female fetus with moderate ventriculomegaly, absence of the cavum septum pellucidum, a dilated third ventricle (Figure 1), echogenic lenses (Figure 2), retrognathia (inferior mandibular angle <50°), hypotelorism (binocular distance at 5^th^ percentile and inter-ocular distance at 50^th^ percentile), and microphthalmia (ocular diameter <5^th^ percentile) (Figure 2).

**Figure 3:**
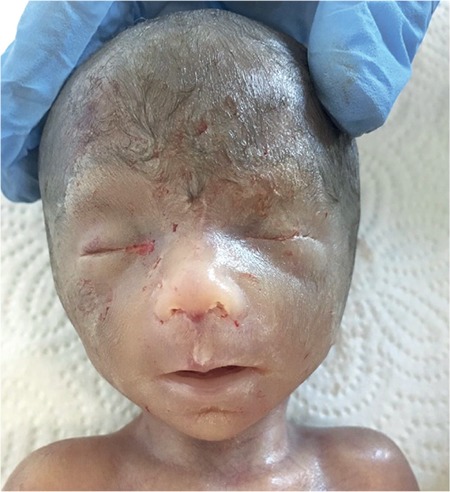
Fetal face: note the narrow palpebral fissures, a long philtrum, cupid’s bow upper lips with a thin vermillion border

**Figure 4:**
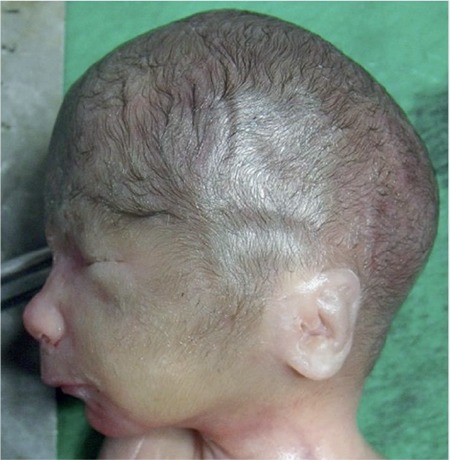
Fascial profile: fascial hirsutism and low-set ears

**Figure 5:**
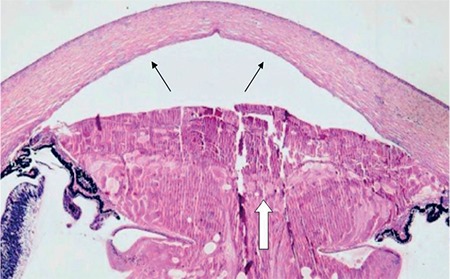
Absence of the posterior corneal stroma and Descemet membrane (black arrows), protrusion of the lens material to the anterior chamber (white arrow)

**Figure 6:**
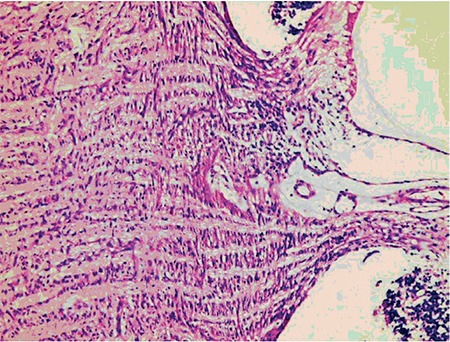
Optic nerve degeneration

Karyotyping and fetal magnetic resonance imaging (MRI) were scheduled owing to findings related to a chromosomal anomaly or a syndrome. Fetal MRI showed agenesis of the corpus callosum, ventriculomegaly, hypotelorism, and bilateral congenital cataracts. Amniocentesis and further karyotyping showed 46, XX chromosomes. Intrauterine fetal death occurred at the 23^rd^ gestational week. A 500-gram female fetus was delivered vaginally after cervical preparation and proper induction. Pathologic autopsy showed narrow palpebral fissures, a long philtrum, cupid’s bow upper lips with a thin vermilion border, and facial hirsutism and low-set ears (Figures 3 and 4), bilateral absence of corneal endothelium and Descemet membrane, bilateral optic nerve degeneration (Figures 5 and 6), bilateral cataracts, agenesis of the corpus callosum, and hydrocephalus.

The autopsy council, including ophthalmologists, confirmed the diagnosis of Peters plus syndrome. The parents were not consanguineous, and their relatives did not indicate a history of such anomalies. Further microarray analyses [Affymetrix, *GRCh37 *(*hg19*)] revealed normal chromosome copy numbers. Analysis of the genes *PAX6* (*11p13*), *PITX2* (or *RIEG1*) (*4p25-26*), *PITX3* (RIEG/PITX homeobox gene family) (*10q25*), *CYP1B1* (cytochrome *P4501B1* gene) (*2p22*), *FKHL7* (Forkhead transcription factor) and *B3GALTL* gene (*13q12.3*) revealed no deletions or duplications in these genes. DNA sequencing would inform us about specific point mutations of these genes that have the potential to play a role in Peters plus syndrome. However, due to the scant amount of DNA in our sample, we could not perform detailed sequencing of these genetic regions.

## Discussion

If typical ocular anomalies of Peters’ anomaly accompany with additional malformations, this situation is referred to as Peters plus syndrome. Peters plus syndrome was initially understood as a causally heterogeneous morphologic entity^(2,3)^. It is now a well-defined syndromic disorder, being confined to a narrower spectrum of accompanying malformations and being related to homozygous or compound heterozygous *B3GALTL* mutations^(7)^. However, other syndromes with a Peter’s anomaly do not fulfill the criteria of Peters plus syndrome.

The clinical features of Peters plus syndrome includes a prominent forehead, narrow palpebral fissures, a long philtrum, cupid’s bow upper lips, cleft lip and palate, preauricular ribs, micrognathia, a broad neck, cataracts and glaucoma, short limbs, brachydactyly, clinodactyly, microcephaly, brain atrophy, agenesis of the corpus callosum, and variable developmental delay and intellectual disability. Some of these features may be present prenatally or at birth, and some may occur at later ages^(2)^.

In contrast to other prenatally detected Peters plus syndrome cases, our case did not show a *B3GALTL* gene mutation. In prenatally detected cases of Peters plus syndrome, including our case, no *B3GALTL* gene mutation has been reported to date^(5,9)^. There may be some explanations for this situation; these cases may be a variant or a phenotypic overlap of Peters plus syndrome^(8)^ or these cases may carry a distinct mutation, which needs further investigation. We also analyzed various mutations in genes that were shown to be linked to the ocular anomaly of Peters’ syndrome or involved in eye development (*PAX6, PITX2, PITX3, CYP1B1, FKHL7*), and no mutations were observed in these genes either. In this situation, our case is more suitable for defining as Peters plus-like syndrome.

Prenatal detection of Peters plus syndrome and like syndromes require a handful of dedicated physicians, including obstetricians, ophthalmologists, genetic specialists, and pathologists. In particular, obstetricians and ultrasonographers should pay attention to the eye, and if ocular anomalies are suspected, craniofacial and skeletal system and fetal growth monitoring should be remembered for the prenatal detection of Peters plus syndrome. In the differential diagnosis of Peters plus syndrome, similar syndromes such as SHORT, Abbruzo-Erickson, GMS, Weill-Marchesani, Michels, Rieger, Walker-Warburg, Cornelia de Lange, Robinow, and fetal alcohol syndrome can be detected according to Orphanet data (http:77www.orpha.net).

Herein, we report a case of Peters plus-like syndrome that was prenatally detected in a patient from Turkey with no family history. The diagnosis was made solely through prenatal ultrasound despite normal fetal chromosomes and no mutation in the *B3GALTL* gene in this sporadic case.

As a result, in cases of fetal anterior segment defects, obstetricians should remember Peter’s anomaly, Peters plus syndrome, and Peters plus-like syndrome, and they should also scan for other accompanying features of these diseases, and perform prenatal invasive tests, including specific gene mutations.
